# Draft Nuclear Genome, Complete Chloroplast Genome, and Complete Mitochondrial Genome for the Biofuel/Bioproduct Feedstock Species *Scenedesmus obliquus* Strain DOE0152z

**DOI:** 10.1128/genomeA.00617-17

**Published:** 2017-08-10

**Authors:** S. R. Starkenburg, J. E. W. Polle, B. Hovde, H. E. Daligault, K. W. Davenport, A. Huang, P. Neofotis, Z. McKie-Krisberg

**Affiliations:** a Bioscience Division, Los Alamos National Laboratory, Los Alamos, New Mexico, USA; b Department of Biology, Brooklyn College of the City University of New York, Brooklyn, New York, USA; c The Graduate Center of the City University of New York, New York, New York, USA

## Abstract

The green alga *Scenedesmus obliquus* is an emerging platform species for the industrial production of biofuels. Here, we report the draft assembly and annotation for the nuclear, plastid, and mitochondrial genomes of *S. obliquus* strain DOE0152z.

## GENOME ANNOUNCEMENT

The coccoid green alga *Scenedesmus obliquus* (Turpin) Kützing, which has the synonyms *Tetradesmus obliquus* (Turpin) Wynne and *Acutodesmus obliquus* (Turpin) Hegewald & Hanagata and the basionym *Achnanthes obliqua* (Turpin), is a common freshwater alga (1, 2) in the family *Scenedesmaceae* within the *Chlorophyta* (= green algae). *S. obliquus* was previously investigated as a feedstock for protein production (3), but the species is now being cultivated for biofuel applications (4–12) and has also been suggested to be a source for edible oils (13).

Purified *S. obliquus* strain DOE0152z genomic DNA was sequenced and assembled using Pacific Biosciences (PacBio) (Menlo Park, CA, USA) long-read sequencing. Briefly, genomic DNA was size selected (20-kb DNA libraries) using Blue Pippin (Sage Science, Beverly, MA, USA) in a 0.75% agarose gel cassette and converted into a 20-kb single-molecule real-time (SMRT) bell library according to the manufacturer’s instructions (PacBio). These size-selected libraries were sequenced on a PacBio RS II single-molecule sequencer (14) utilizing C3-P5 chemistry and 3-h movies. The library preparation protocol was repeated twice to generate two libraries/sequencing templates from the same genomic DNA pool. In total, 46 SMRT cells were sequenced, and these cells collectively produced 556,064 subreads with a mean subread length of 5,094 kb, which provided 18,115 Mbp of data or approximately 86× coverage of the assembled genome size. All PacBio subreads greater than 5 kb in length were assembled with HGAP version 2.3.0 (15). The mitochondrial and chloroplast genomes were assembled manually from fragmented contigs pulled from this initial assembly by mapping to previously sequenced *Scenedesmus* organellar genomes (GenBank numbers AF204057 and DQ396875, respectively). Chloroplast and mitochondrial genomes were extracted with DECONseq software, using a database constructed from previously published mitochondrial sequences for *S. obliquus* strain UTEX 78, and chloroplast sequences from *A. obliquus* (GenBank accession number DQ396875). Extracted contigs were mapped back onto reference sequences to ensure full coverage to plastid and mitochondrial genomes.

The final genome assembly resulted in 2,705 contigs with an *N*_50_ of 155,544 bp, a minimum contig size of 11,140 bp, a maximum contig size of 2,334,183 bp, and a total assembly size of 207,967,116 bp. Recently, a draft assembly of the nuclear genome was reported for *Tetradesmus obliquus* strain UTEX393 with a size of approximately 109 Mbp (16). In contrast, the draft assembly of the nuclear genome of DOE0152z is almost twice the size at >210 Mbp. Furthermore, strain DOE0152z contains a plastid chromosome of 167,272 bp and the mitochondrial genome assembled to 41,704 bp. To the best of our knowledge, this is the first report of a nuclear, plastid, and mitochondrial genome assembly from the same *Scenedesmus* strain.

### Accession number(s).

This whole-genome project is publicly available on the LANL Greenhouse page (https://greenhouse.lanl.gov/greenhouse/) and was deposited in DDBJ/ENA/GenBank under the accession number NEDT00000000. The version described in this paper is version NEDT01000000.
